# Association Between Early Pain and Miniscrew-Related Oral Impact After Orthodontic Miniscrew Placement: A Prospective Cohort Study

**DOI:** 10.3390/jcm15207852

**Published:** 2026-10-12

**Authors:** Hadi M. Ismail

**Affiliations:** Department of Orthodontics, College of Dentistry, University of Sulaimani, Sulaimani P.O. Box 1124-30, Iraq; hadi.ismail@univsul.edu.iq; Tel.: +964-7702106211

**Keywords:** orthodontic miniscrews, skeletal anchorage, temporary anchorage device, pain, miniscrew-related oral impact, patient-reported outcomes

## Abstract

**Background/Objectives:** Evidence integrating patient-reported outcomes with the clinical performance of orthodontic miniscrews remains limited. This study evaluated pain, miniscrew-related oral impact, and six-month clinical outcomes following miniscrew placement. **Methods:** In this single-centre prospective observational cohort, 111 patients were screened, 104 were enrolled, and 100 patients receiving 270 clinically indicated miniscrews contributed complete data. Pain was assessed using a visual analogue scale immediately after placement, at 24 h, and at one week. A preliminary eight-item miniscrew-related oral-impact questionnaire was completed at one week. Analyses included the Friedman test with Holm-adjusted pairwise comparisons, Spearman correlations, and logistic generalised estimating equations (GEEs) accounting for within-patient clustering. **Results:** Median pain decreased from 6.0 (IQR, 3–8) immediately after placement to 4.0 (IQR, 0–7.25) at 24 h and 0.0 (IQR, 0–2) at one week (*p* < 0.001; Kendall’s *W* = 0.568; pairwise *r* = 0.366–0.758). Analgesics were used by 57% of participants. The mean oral-impact score was 6.08 ± 5.92 on a 0–32 scale. Greater immediate post-placement pain was associated with higher oral-impact scores (*ρ* = 0.431; *p* < 0.001). At six months, 250 of 270 miniscrews were successful (92.6%). At the miniscrew level, soft-tissue injury and localised inflammation affected 7.8% and 4.4%, respectively. No examined factor was reliably associated with failure in the clustered GEE model. At the patient level, 78% were satisfied or very satisfied, and 92% would recommend the procedure. **Conclusions:** Pain decreased substantially within one week, while greater early pain was associated with less favourable oral-impact scores. A 92.6% six-month success rate and favourable patient acceptance were observed. The questionnaire requires further validation, and the exploratory failure estimates require confirmation.

## 1. Introduction

Orthodontic miniscrews provide temporary skeletal anchorage without relying on patient compliance and facilitate movements such as en-masse retraction, intrusion, and distalisation [1,2,3,4,5]. Reported success rates generally range from 80% to 95% [6,7,8]; however, postoperative pain, peri-miniscrew inflammation, loosening, and failure may occur [9].

Increasing attention has been given to patient-reported outcomes in orthodontics. Pain associated with orthodontic procedures, including miniscrew placement, is a common patient concern and may influence treatment acceptance and satisfaction. Available evidence indicates that discomfort is usually greatest during the first 24–48 h and diminishes during the first postoperative week [9,10,11,12,13,14,15]. However, previous studies have generally examined discomfort, oral impact, or miniscrew stability separately. Prospective evidence linking early pain with one-week miniscrew-related oral impact while concurrently documenting clinical performance in the same cohort remains limited [10,12,14,15].

Beyond pain, orthodontic procedures may influence patients’ oral health-related quality of life [10,12]. Nevertheless, generic oral health-related quality-of-life (OHRQoL) instruments may not adequately capture the specific short-term experiences associated with orthodontic miniscrew placement. Therefore, the present study used a preliminary eight-item miniscrew-related oral-impact questionnaire designed to assess procedure-specific effects during the first postoperative week. The items covered pain, mucosal irritation, difficulty chewing, speech difficulty, embarrassment, reduced confidence, avoidance of smiling or social interaction, and sleep disturbance. These items represented intended physical, functional, psychological, and social content areas; however, these groupings were conceptual and were not evaluated as validated subscales. Although the questionnaire was developed with conceptual reference to relevant Oral Health Impact Profile (OHIP) domains, the resulting instrument was neither a validated adaptation nor a shortened version of the OHIP and should not be interpreted as a validated oral health-related quality-of-life measure. Its total score was therefore treated as an exploratory measure of miniscrew-related oral impact.

This study addresses the identified evidence gap by integrating repeated assessments of early pain, a procedure-specific oral-impact assessment, and six-month clinical miniscrew outcomes within the same prospective cohort. It also accounts for the clustering of multiple miniscrews within individual patients and includes a sensitivity analysis excluding the questionnaire’s pain item. Accordingly, the primary aim was to evaluate pain intensity, miniscrew-related oral impact, and six-month clinical outcomes following orthodontic miniscrew placement. A secondary objective was to examine the associations between pain recorded immediately after placement, at 24 h, and at one week, and the total oral-impact score recorded at one week. We hypothesised that greater procedural and early postoperative pain would be associated with higher, and therefore less favourable, oral-impact scores.

## 2. Materials and Methods

### 2.1. Study Design and Setting

This prospective observational cohort study evaluated patient-reported outcomes and six-month clinical outcomes of orthodontic miniscrews placed in patients undergoing fixed orthodontic treatment at City Smile Design dental centre, a private dental clinic. The study was conducted in accordance with the Declaration of Helsinki and was approved by the Ethics Committee of the College of Dentistry, University of Sulaimani (approval number COD-EC-25-0157; 16 November 2025). Participants were recruited consecutively between 18 November and 30 December 2025. Each miniscrew was followed for six months from its placement date, and the final six-month assessment was completed on 30 June 2026. All patients received both oral and written information regarding the treatment plan, the clinical study, and the use of miniscrews during the treatment procedure. Written informed consent was subsequently obtained from all participants, and from parents or guardians in the case of minors. The decision to use miniscrew anchorage was made solely according to each patient’s orthodontic treatment requirements and preceded the decision regarding research participation. No miniscrew was placed solely for the purposes of this study, and declining research participation did not affect the patient’s treatment.

All eligible patients receiving orthodontic miniscrews during the study period were consecutively recruited to reduce selection bias. No miniscrew was electively removed because the intended orthodontic movement had been completed before the six-month assessment. Miniscrews were removed earlier only when clinically significant mobility or loosening caused loss of anchorage function and required removal or replacement.

### 2.2. Patient Selection

Eligible participants were patients undergoing fixed-appliance orthodontic treatment who required at least one miniscrew for clinically indicated orthodontic anchorage. Baseline exclusion criteria were miniscrews used for non-orthodontic purposes, systemic conditions likely to affect bone metabolism, medications known to influence bone remodelling, active periodontal disease, or oral hygiene considered clinically inadequate for miniscrew placement. Follow-up completeness was not considered a baseline eligibility criterion. Participants without the relevant outcome assessment were recorded as lost to follow-up and handled as described in the statistical analysis.

### 2.3. Sample Size Calculation

Sample size calculation was based on a patient-level analysis of the primary association between early pain and the total score on the preliminary eight-item miniscrew-related oral-impact questionnaire. Previous orthodontic studies have demonstrated a significant relationship between pain intensity and oral health-related quality of life during treatment [13,14]. For planning purposes, a conservative small-to-moderate correlation coefficient of *r* = 0.28 was therefore assumed for sample-size planning rather than derived directly from a single published estimate. Using Fisher’s (z)-transformation, a two-sided significance level of 0.05 and 80% statistical power indicated that at least 98 patients with complete data were required. No fixed a priori attrition percentage was specified in the study protocol; recruitment therefore continued pragmatically beyond the calculated minimum to preserve the requirement for at least 98 complete cases. Of the 104 participants enrolled, one lacked complete baseline measurements and three were lost before the one-week assessment, leaving 100 complete cases for the primary patient-level analysis. The 270 miniscrews received by these participants contributed to the separate screw-level descriptive and failure analyses.

### 2.4. Clinical Procedure

Placement sites were categorised by arch and interradicular region:

- Upper arch: premolar region (between first and second premolar, anterior to molar), and molar region (between first and second molar).
- Lower arch: premolar region (between first and second premolar, anterior to molar), and molar region (between first and second molar).

All participants were treated by the investigator (H.M.I.). Fixed appliances used a 0.022-inch slot Roth prescription (Signature; Ortho-Care Ltd., West Yorkshire, UK), with a recommended archwire sequence of 0.014-inch nickel–titanium, 0.019 × 0.025-inch nickel–titanium, and 0.019 × 0.025-inch stainless steel with posts.

**Figure 1:**
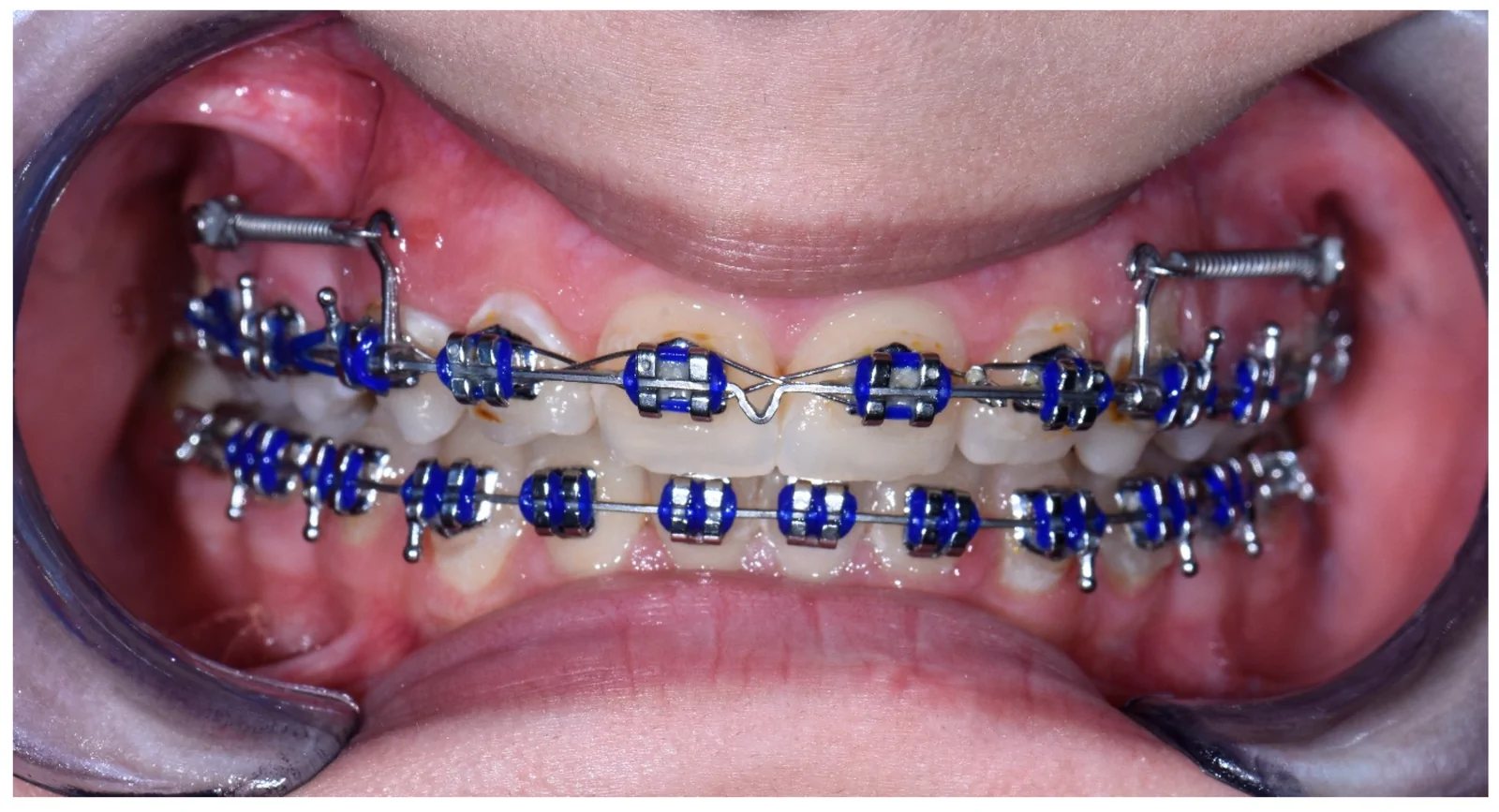
Clinical photograph showing an interradicular orthodontic miniscrew under immediate loading with a nickel–titanium closed-coil spring.

All miniscrews were placed by the investigator (H.M.I.), who had 10 years of miniscrew-placement experience, according to the following protocol:

1. Topical anaesthesia with 5% lidocaine gel placed in the area of the miniscrew site.

2. Buccal infiltration of 0.3 mL Xylocaine Dental Adrenalin per site (lidocaine hydrochloride 20 mg/mL, adrenaline 12.5 µg/mL; Dentsply Pharmaceutical) delivered using an insulin syringe.

3. A chlorhexidine-containing mouth rinse was used for 60 s (KIN Gingival Complex; Laboratorios KIN, Barcelona, Spain).

4. Miniscrews (GNI Co., Ltd., Gyeonggi-do, Republic of Korea, or Azdent, Henan Baistra Industries Corp., Henan, China) were inserted interdentally. Insertion torque was not measured. Insertion angle and loading force were selected clinically but were not recorded as continuous variables.

5. Periapical radiographs were taken when clinically indicated to evaluate screw position and to rule out possible root proximity.

6. Miniscrews were loaded immediately as direct anchorage using nickel–titanium coil springs or elastomeric chains according to the required treatment mechanics, as illustrated in Figure 1.

7. Participants were advised that paracetamol could be used when clinically required, in accordance with age-appropriate prescribing instructions. Analgesic use during the first postoperative week was recorded as a binary variable (yes/no). Medication type, dose, frequency, timing relative to each pain assessment, and indication for use were neither recorded nor standardised. Accordingly, analgesic-related analyses were considered exploratory and interpreted as associations potentially affected by confounding by indication, rather than as estimates of treatment effects.

### 2.5. Preliminary Questionnaire Development and Reliability Assessment

The preliminary eight-item miniscrew-related oral-impact questionnaire was developed to assess the physical, functional, psychological, and social impacts of orthodontic miniscrew placement. The eight-item questionnaire was conceptually informed by OHIP domains but did not constitute a shortened or formally validated version of the OHIP-14. Items were rewritten to address miniscrew-specific experiences, including pain at the insertion site, chewing difficulty, mucosal irritation, speech difficulty, embarrassment related to screw visibility, avoidance of smiling or social interaction, sleep disturbance, and reduced confidence. The wording was reviewed for local comprehensibility by orthodontists and patients. No formal cross-cultural adaptation or factor analysis was performed. The preliminary evaluation of the adapted instrument comprised preliminary content review and reliability assessment.

#### 2.5.1. Preliminary Content Review (Qualitative Assessment)

Three orthodontists independently reviewed the draft items for clarity and relevance of the questions to the intended domains. In addition, five orthodontic patients (not included in the main study) reviewed the questionnaire to assess how clear and understandable the wording was. This process supported face validity and preliminary content relevance; a formal content-validity index was not calculated. Based on their feedback, minor changes were made. The final questionnaire comprised eight items organised into four intended conceptual groupings: physical, functional, psychological, and social. These groupings were not evaluated as validated subscales.

#### 2.5.2. Reliability Assessment (Quantitative Assessment)

Test–retest reproducibility was examined in 10 orthodontic patients, not included in the main study, who completed the final eight-item questionnaire twice at a two-week interval. Reproducibility was estimated using a two-way random-effects, single-measurement, absolute-agreement intraclass correlation coefficient [ICC(2,1)] with a 95% confidence interval. Because the test–retest sample was small and the instrument had not undergone assessments of construct validity, dimensionality, or responsiveness, the psychometric findings were considered preliminary.

Internal consistency was assessed in the main study sample (*n* = 100) using Cronbach’s alpha and McDonald’s omega. Test–retest reproducibility was evaluated in the separate pilot sample using ICC(2,1), and systematic test–retest differences were examined using the Wilcoxon signed-rank test.

### 2.6. Measurements

#### 2.6.1. Primary Outcome

Pain intensity was assessed prospectively at three predefined time points. Immediately after miniscrew placement, participants rated the pain experienced during insertion using a 10 cm visual analogue scale ranging from 0 (“no pain”) to 10 (“worst imaginable pain”). Current pain intensity was subsequently recorded at 24 h and one week after placement.

At the one-week assessment, participants completed the preliminary eight-item miniscrew-related oral-impact questionnaire, reporting the frequency of impacts experienced during the first postoperative week. Participants were instructed to report the frequency of each impact over the entire first postoperative week rather than only their condition at the time of questionnaire completion. Because the questionnaire was administered at a single time point, it provided a retrospective summary of first-week oral impact and was not intended to measure longitudinal change. The items assessed the frequency of miniscrew-related oral impacts during the first postoperative week and were rated on a 5-point Likert scale, with possible scores of 0: “Never”; 1: “Rarely”; 2: “Sometimes”; 3: “Often”; and 4: “Always”. The sum resulted in a total score between 0 and 32, with higher scores indicating more frequent reported impact.

#### 2.6.2. Secondary Outcomes

Miniscrew success was assessed over the predefined six-month observation period, irrespective of whether the patient’s overall orthodontic treatment had been completed. Success was defined as clinical stability and continued functional anchorage without removal or replacement because of mobility, infection, soft-tissue complications, or loss of anchorage during the six-month period. No miniscrew was electively removed because of completion of the intended orthodontic movement before the six-month assessment. Successful miniscrews were retained for the complete observation period and were subsequently removed when their intended orthodontic purpose had been achieved.

Miniscrew failure was defined as clinical mobility, loosening, or loss of anchorage that required removal or replacement of the screw before completion of the intended orthodontic mechanics.

Complications included miniscrew loosening, soft-tissue injury, and localised peri-miniscrew inflammation. Localised inflammation was defined as clinically observed erythema and/or swelling of the surrounding soft tissues, with or without bleeding or tenderness. Soft-tissue injury and localised inflammation were recorded separately and were not classified as failures unless they resulted in loss of miniscrew stability or function.

At the predefined six-month study assessment, while orthodontic treatment remained ongoing for most participants, satisfaction and willingness to recommend miniscrew therapy were recorded.

Overall patient satisfaction: Assessed on a 5-point Likert-type scale evaluating the overall treatment experience (very dissatisfied, dissatisfied, neutral, satisfied, and very satisfied).

Willingness to recommend: Evaluated as a binary indicator (yes/no) asking whether patients would recommend miniscrew therapy to other orthodontic patients.

Patient-reported outcomes, including pain, oral-impact scores, analgesic use, satisfaction, and willingness to recommend, are reported per patient (denominator *n* = 100). Miniscrew success, failure, and complications are reported per screw (denominator *n* = 270). Failure-predictor analyses used the screw as the observation unit and accounted for clustering within patients using a logistic generalised estimating equation (GEE).

### 2.7. Statistical Analysis

Data were analysed using IBM SPSS Statistics, version 26.0 (IBM Corp., Armonk, NY, USA), with McDonald’s omega calculated via the Hayes OMEGA macro extension. All tests were two-sided, with statistical significance set at *p* < 0.05. Continuous variables were summarised as mean ± standard deviation or median and interquartile range, as appropriate, and categorical variables as frequencies and percentages. Distributional assumptions were assessed using the Shapiro–Wilk test.

Internal consistency of the preliminary eight-item miniscrew-related oral-impact questionnaire was evaluated in the main sample (*N* = 100) using Cronbach’s alpha and McDonald’s omega. Test–retest reproducibility was assessed in a separate pilot sample (*n* = 10) using a two-way random-effects, single-measurement, absolute-agreement intraclass correlation coefficient [ICC(2,1)] with a 95% confidence interval. Systematic test–retest differences were examined using the Wilcoxon signed-rank test.

Changes in pain across placement, 24 h, and one week were analysed using the Friedman test, with Kendall’s *W* reported as the overall effect size. Significant results were followed by pairwise Wilcoxon signed-rank tests with Holm adjustment, and effect sizes were expressed as *r*. Pain scores were compared between analgesic users and non-users using the Mann–Whitney *U* test.

Associations between pain at each assessment point and the total oral-impact score were assessed using Spearman’s rank correlation coefficient (*ρ*). To address potential part–whole correlation, these analyses were repeated using a modified seven-item oral-impact score excluding the pain/discomfort item. Additional sensitivity analyses included stratified Spearman correlations according to analgesic use and multivariable linear regression with the one-week total oral-impact score as the dependent variable and placement pain and analgesic use as predictors. Unstandardised coefficients (*B*), standard errors, standardised coefficients (β), 95% confidence intervals, *p*-values, *R*^2^, and adjusted *R*^2^ were reported. Analgesic-related findings were interpreted as adjusted associations rather than treatment effects because analgesic use was not randomised or standardised.

Patient-reported outcomes were analysed at the patient level, whereas miniscrew outcomes were analysed at the screw level. Miniscrew failure was analysed using a logistic generalised estimating equation (GEE) model with a binomial distribution, logit link, exchangeable working correlation structure, patient identifier as the clustering variable, and robust sandwich standard errors. Anatomical arch, miniscrew length, miniscrew diameter, and patient age were selected a priori on clinical grounds and entered simultaneously; no automated variable-selection procedure was used. Adjusted odds ratios (ORs), robust 95% confidence intervals, and *p*-values were reported. Because only 20 failures occurred—approximately five events per estimated predictor coefficient—the model was considered insufficiently supported for confirmatory inference. The analysis was therefore treated as exploratory, with interpretation emphasising the magnitude and precision of the estimates rather than statistical significance alone.

## 3. Results

### 3.1. Sample Characteristics

**Figure 2:**
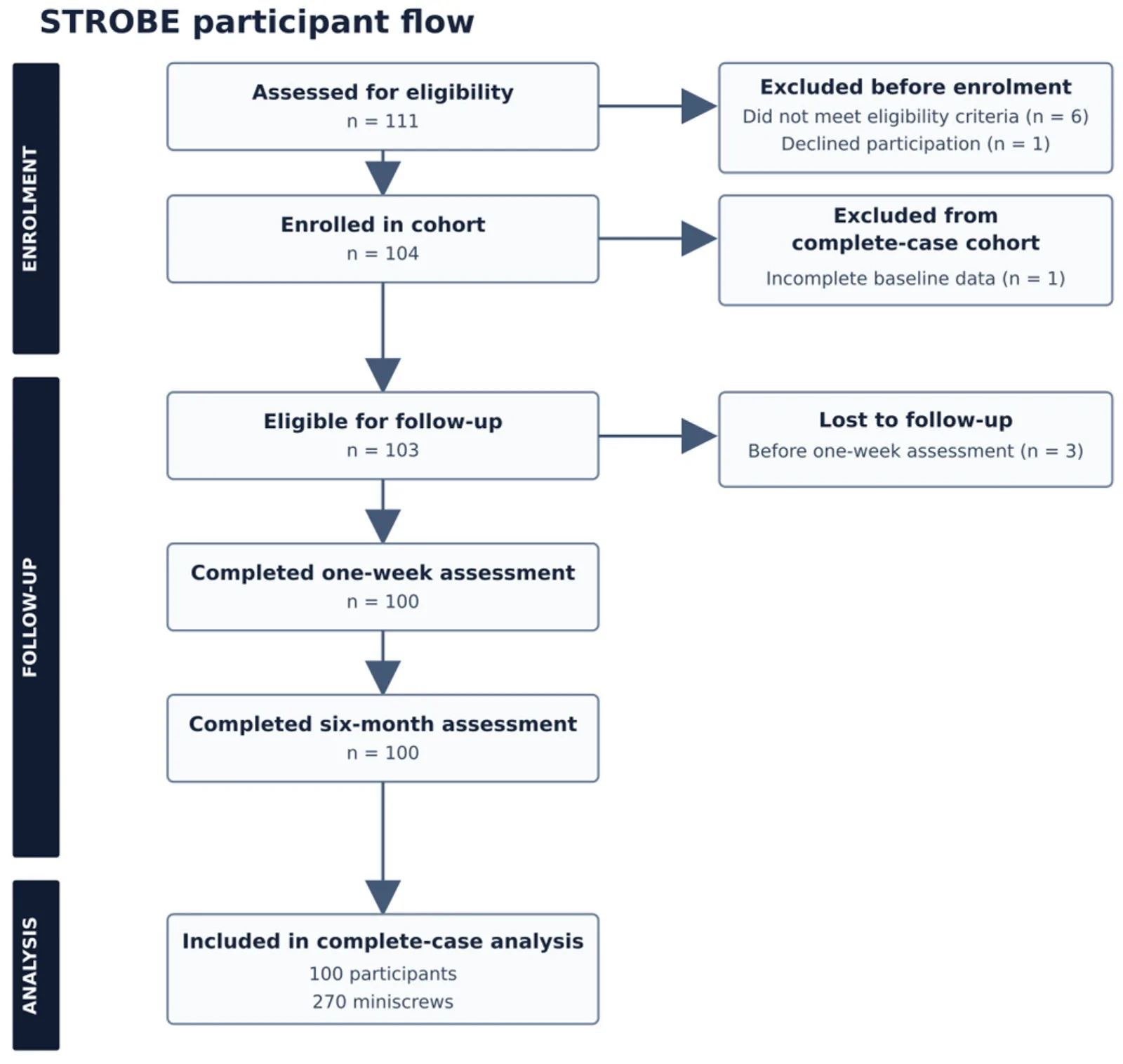
Participant flow through the study.

A total of 111 individuals were assessed for eligibility. Six did not meet the eligibility criteria and one declined participation; therefore, 104 participants were enrolled. One participant lacked complete baseline measurements and was excluded before follow-up, leaving 103 participants eligible for follow-up. Three participants were lost before the one-week assessment. The final complete-case analytical cohort comprised 100 participants with 270 miniscrews, all of whom had recorded six-month outcomes. No missing outcomes were imputed. Participant flow is presented in Figure 2.

The analytical cohort comprised 100 participants who received 270 orthodontic miniscrews (mean, 2.70 ± 1.26 screws per participant; range, 1–7). The median participant age was 21.0 years (IQR, 17.0–27.0; mean, 22.4 ± 7.4 years; range, 14–52 years). The sample comprised 71 women (71.0%) and 29 men (29.0%). The median miniscrew length was 10.0 mm (IQR, 8.0–10.0; mean, 9.33 ± 0.94 mm); 90 screws (33.3%) were 8.0 mm and 180 (66.7%) were 10.0 mm long. The median diameter was 1.6 mm (IQR, 1.6–1.6; mean, 1.56 ± 0.08 mm); 60 screws (22.2%) had a diameter of 1.4 mm and 210 (77.8%) had a diameter of 1.6 mm. Miniscrews were placed at interradicular sites in the maxilla and mandible according to treatment requirements; their anatomical distribution is presented in Table 1.

### 3.2. Preliminary Evaluation and Reliability of the Questionnaire

#### 3.2.1. Preliminary Content Review

Review by three orthodontists and five orthodontic patients indicated that the items were understandable and relevant to the intended miniscrew-related oral impacts. Minor wording revisions were made. This preliminary review supported item clarity and content relevance but did not constitute full psychometric validation or confirmation of the proposed domain structure.

#### 3.2.2. Reliability

Test–retest reliability evaluated on a pilot subsample (*n* = 10) using a two-way random-effects single-measurement model [ICC(2,1)] showed high test–retest reproducibility in the small pilot sample (ICC = 0.92, 95% CI = 0.72–0.98, *p* < 0.001). A Wilcoxon signed-rank test revealed no significant difference between test and retest total scores (*p* = 0.642). In the full patient sample (*N* = 100), internal consistency was acceptable, yielding a Cronbach’s alpha of α = 0.716 and a McDonald’s omega of ω = 0.803, as presented in Table 2.

### 3.3. Pain Assessment

**Figure 3:**
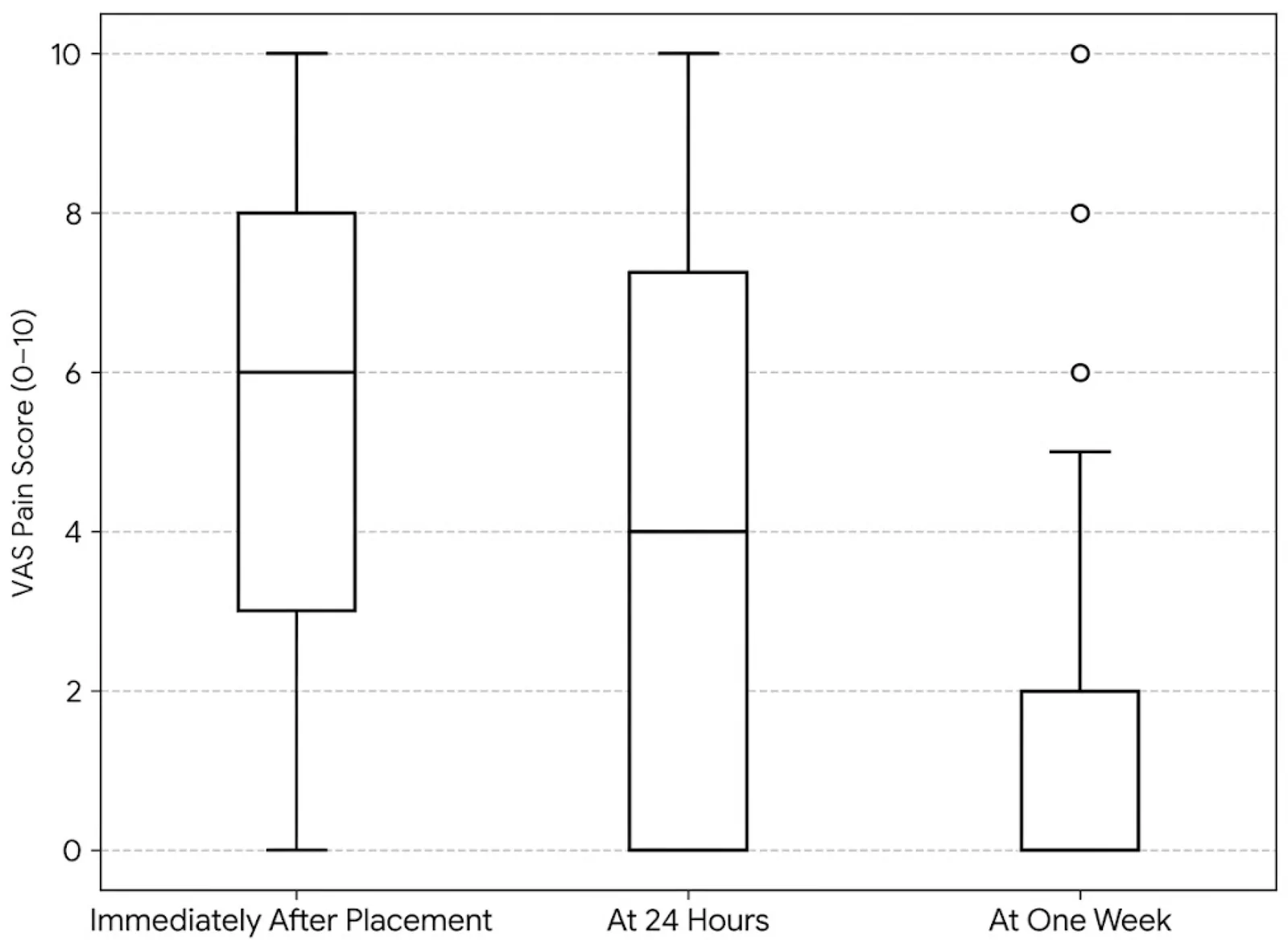
Box plot illustrating the distribution of pain intensity scores (VAS 0–10) at three time points: immediately after miniscrew placement, 24 h post-insertion, and one week after placement. The median is the horizontal line within each box, and the boxes indicate the interquartile range (25th–75th percentiles). Whiskers extend to maximum/minimum non-outlier values, and dots represent individual outliers.

Pain differed across the three assessment points (χ^2^(2) = 113.64, *p* < 0.001; Kendall’s *W* = 0.568). Holm-adjusted Wilcoxon signed-rank comparisons demonstrated reductions from immediately after placement to 24 h (*W* = 861.0, adjusted *p* < 0.001, effect size *r* = 0.366), immediately after placement to one week (*W* = 155.0, adjusted *p* < 0.001, effect size *r* = 0.758), and 24 h to one week (*W* = 0.0, adjusted *p* < 0.001, effect size *r* = 0.713). Median pain scores were 6.0 (IQR 3.0–8.0), 4.0 (IQR 0.0–7.25), and 0.0 (IQR 0.0–2.0), respectively, as shown in Figure 3.

Analgesic use was reported by 57.0% of patients (*n* = 57). Mann–Whitney U tests showed that analgesic users reported significantly higher pain than non-users at 24 h (Median = 6.0, IQR = 2.0–8.0 vs. Median = 3.0, IQR = 0.0–6.5; U = 1619.5, *p* = 0.005) and at one week (Median = 0.0, IQR = 0.0–3.0 vs. Median = 0.0, IQR = 0.0–0.0; U = 1472.5, *p* = 0.034). This pattern is consistent with confounding by indication, whereby patients experiencing greater pain may have been more likely to use analgesics. These comparisons should not be interpreted as estimates of analgesic effectiveness.

Because participants with greater pain may have been more likely to take analgesics, a secondary sensitivity analysis examined the association between pain intensity and the total oral-impact score while accounting for reported analgesic use (57% users vs. 43% non-users).

Stratified Spearman rank correlations showed that procedural pain during placement remained significantly associated with miniscrew-related oral impact impairment in both analgesic users (*ρ* = 0.350, *p* = 0.008) and non-users (*ρ* = 0.412, *p* = 0.006). In an exploratory linear regression model, immediate post-placement pain remained associated with the one-week total oral-impact score after inclusion of analgesic use (B = 0.640, SE = 0.180, standardised β = 0.336, 95% CI: 0.283 to 0.996, *p* = 0.001). Analgesic use was also associated with the total oral-impact score (B = 2.534, SE = 1.131, standardised β = 0.211, 95% CI: 0.288 to 4.780, *p* = 0.027). The model explained 19.4% of the variance (adjusted R^2^ = 17.8%). Because analgesic use was self-selected and its timing and dose were not standardised, this sensitivity model does not estimate an analgesic treatment effect.

### 3.4. Miniscrew-Related Oral-Impact Outcomes

**Figure 4:**
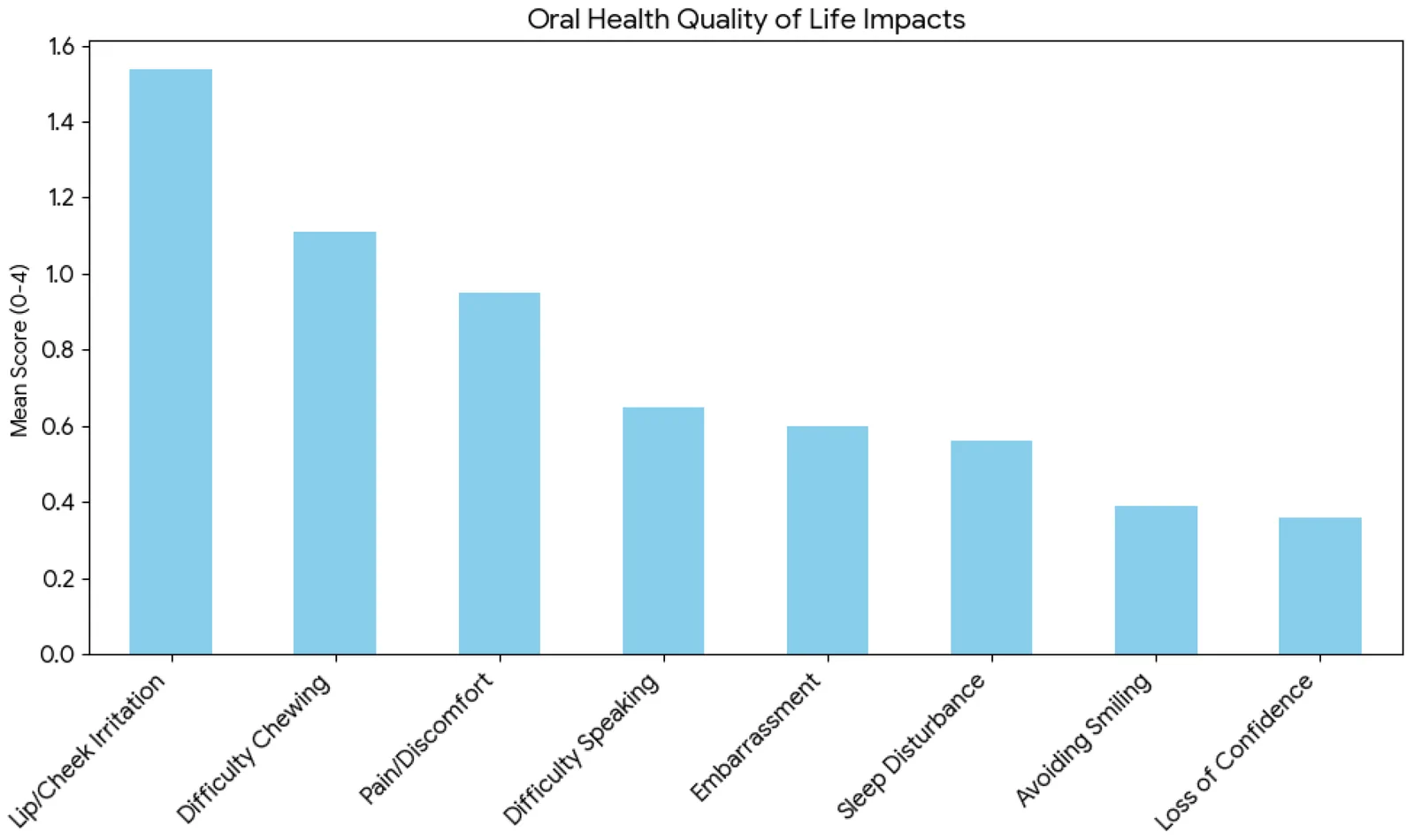
Mean item scores on the preliminary eight-item miniscrew-related oral-impact questionnaire at one week. Items were scored from 0 (never) to 4 (always); higher scores indicate more frequent reported impact.

The mean total oral-impact score was 6.08 ± 5.92. Scores were concentrated toward the lower end of the instrument; however, no validated severity threshold or minimally important difference is available. Soft-tissue irritation and chewing difficulty were the most frequently reported experiences. Psychological and social items generally had lower scores. Detailed patient-reported oral impacts are presented in Table 3 and Figure 4.

A significant positive correlation was observed between pain levels and oral impact across all time points. The strongest association was found between pain experienced during placement and the total oral impact score (*ρ* = 0.431, *p* < 0.001). Significant, though slightly weaker, correlations were also identified for pain at 24 h (*ρ* = 0.297, *p* = 0.003) and at one week (*ρ* = 0.312, *p* = 0.002). These results indicate that greater pain intensity was associated with more frequent miniscrew-related oral impacts, as shown in Table 4.

To confirm that the observed associations between pain and oral impact were not driven solely by item-overlap bias (i.e., local pain being measured both as an independent predictor and within the miniscrew-related oral impact via Item 1), a sensitivity analysis was conducted using a modified seven-item oral-impact score excluding Item 1, as shown in Table 3.

The modified seven-item oral-impact score yielded a median of 3.5 (IQR 1.00–8.00; mean 5.23 ± 5.24). Spearman rank correlations confirmed that acute pain intensity across all time points remained significantly correlated with overall oral-impact impairment independent of direct pain reporting: placement pain (*ρ* = 0.395, *p* < 0.001 ***), 24 h pain (*ρ* = 0.331, *p* < 0.001 ***), and one-week pain (*ρ* = 0.346, *p* < 0.001 ***). These findings indicate that initial procedural and postoperative pain remained associated with the modified score comprising the seven non-pain oral-impact items (such as chewing, speech, and daily activities).

### 3.5. Miniscrew Stability and Failure Rate

Among the 270 miniscrews placed, 20 failures were recorded, corresponding to a failure rate of 7.4% and an overall success rate of 92.6% under immediate orthodontic loading. Success rates by arch and specific placement sites are summarised in Table 1.

To account for the clustering of 270 miniscrews within 100 patients, a binary logistic GEE model with an exchangeable working correlation structure and robust standard errors was fitted (Table 5). No examined factor was reliably associated with miniscrew failure: anatomical arch (maxilla versus mandible: adjusted OR = 0.63, 95% CI: 0.22–1.82; *p* = 0.386), miniscrew length per 1 mm increase (adjusted OR = 0.92, 95% CI: 0.77–1.09; *p* = 0.315), miniscrew diameter per 1 mm increase (adjusted OR = 0.42, 95% CI: 0.03–5.76; *p* = 0.551), or patient age per one-year increase (adjusted OR = 1.01, 95% CI: 0.95–1.08; *p* = 0.654). All confidence intervals included the null value, and the particularly wide interval for miniscrew diameter indicated substantial imprecision. Given the limited number of failures relative to the four simultaneously modelled predictors, these estimates should be interpreted as exploratory rather than as evidence confirming or excluding clinically relevant associations.

### 3.6. Complications

A total of 20 (7.4%) screws experienced loosening (failure). Soft-tissue injury was recorded for 21 of 270 miniscrews (7.8%). Soft-tissue injuries occurred most commonly adjacent to retraction springs in the maxillary canine area and around mandibular molar miniscrews. Other complications included local inflammation in 12 out of 270 miniscrews (4.4%). No miniscrew fracture occurred during insertion, loading, or removal.

### 3.7. Patient Satisfaction and Willingness to Recommend

Overall patient satisfaction with miniscrew treatment was generally favourable. At six months, 39 patients were very satisfied, 39 were satisfied, 19 were neutral, and three were dissatisfied; no participant reported being very dissatisfied. Overall, 78 patients (78%) were satisfied or very satisfied, and 92 (92%) stated that they would recommend the procedure.

## 4. Discussion

This single-centre prospective observational cohort produced three principal findings. First, pain decreased substantially from immediately after miniscrew placement to one week, with a large overall time effect. Second, greater early pain was associated with less favourable scores on the preliminary miniscrew-related oral-impact questionnaire administered at one week, including after exclusion of the questionnaire’s pain item; however, these associations were modest, temporally overlapping, and observational. Third, 92.6% of the 270 clinically indicated miniscrews remained successful at six months, accompanied by generally favourable patient satisfaction and willingness to recommend the procedure. The exploratory regression analysis did not identify reliable factors associated with failure, although its precision was limited by the small number of failure events.

The median pain rating decreased from 6.0 immediately after placement to 4.0 at 24 h and 0.0 at one week. The large overall effect size indicates that this reduction was substantial within the study sample. This trajectory is broadly consistent with previous evidence showing that discomfort associated with miniscrew insertion is concentrated in the early postoperative period and generally diminishes within several days [9,10,14,15,16]. Differences in reported pain magnitude among studies may reflect variations in anaesthetic protocols, insertion techniques, placement sites, the number of miniscrews placed, patient characteristics, and the timing of pain assessment. Patients may therefore be advised that discomfort usually decreases rapidly, while recognising that individual experiences vary.

Analgesics were used by 57% of participants. However, their use was self-selected and recorded only as any use during the first postoperative week. Medication type, dose, frequency, timing relative to pain assessment, and indication were not recorded or standardised. Higher pain among analgesic users is therefore most plausibly explained by confounding by indication, whereby participants experiencing greater pain were more likely to take medication. These exploratory comparisons cannot be interpreted as estimates of analgesic effectiveness or harm.

Greater immediate post-placement pain was associated with a less favourable oral-impact score at one week, whereas associations with pain at 24 h and one week were weaker. These findings suggest that participants reporting greater early pain also tended to report greater physical, functional, psychological, or social impact during the first postoperative week. Nevertheless, the correlations were weak-to-moderate and cannot establish that pain caused the reported oral impacts. Previous studies have similarly indicated that pain and discomfort may adversely affect short-term patient-reported outcomes during orthodontic treatment [13,15,17].

The questionnaire scores were concentrated toward the lower end of the 0–32 scale. However, this finding should remain descriptive because no validated severity threshold or minimally important difference has been established for this preliminary instrument. It would therefore be inappropriate to classify the observed scores as “minimal,” “mild,” or clinically unimportant. At the item level, soft-tissue irritation and difficulty chewing were among the more frequently reported experiences, indicating the importance of appropriate oral-hygiene advice, soft-tissue monitoring, and clinical review when symptoms persist.

The association between pain and oral impact remained after the pain/discomfort item was excluded from the questionnaire score, reducing concern that the finding resulted solely from direct part–whole correlation. Nevertheless, the sensitivity analysis did not eliminate recall bias, common-method bias, or temporal overlap. The questionnaire was completed at one week and retrospectively assessed experiences during the same postoperative period in which pain was recorded. This shared assessment period may have strengthened the observed associations. Furthermore, because oral impact was evaluated only at one week, the study did not assess longitudinal changes in oral impact or oral health-related quality of life. Although previous evidence suggests that orthodontic discomfort may diminish with adaptation [14,16,17], such a trajectory was not evaluated in this cohort.

Cronbach’s alpha and McDonald’s omega indicated acceptable internal consistency, while the small pilot assessment indicated high test–retest reproducibility. These findings provide preliminary evidence of score reliability but do not establish instrument validity. Construct validity, dimensionality, responsiveness, measurement invariance, cross-cultural validity, and a minimally important difference were not evaluated. The intended conceptual groupings must not be interpreted as validated subscales. Accordingly, the instrument should be described as a preliminary miniscrew-related oral-impact questionnaire rather than as a validated oral health-related quality-of-life measure. Further development and independent validation should incorporate established patient-reported outcome measure methodology, including appropriate assessment of content, construct, and cross-cultural validity [18,19,20].

At the miniscrew level, 250 of 270 miniscrews remained functionally successful at six months, corresponding to a success proportion of 92.6%. This finding lies within previously reported ranges [6,21,22,23] and describes miniscrew performance under the clinical protocol and case mix of the present cohort. It should not be interpreted as evidence of universal miniscrew reliability. Soft-tissue injury and localised inflammation affected 7.8% and 4.4% of miniscrews, respectively. These percentages represent screw-level outcomes and should not be interpreted as the proportions of patients experiencing these complications.

The clustered logistic GEE model did not identify reliable associations between the examined factors and miniscrew failure. However, absence of statistical significance does not demonstrate absence of clinically relevant associations. Only 20 failures occurred, while four predictors were entered simultaneously into the model, resulting in limited statistical power, wide confidence intervals, and potentially unstable estimates. Although GEE appropriately accounted for multiple miniscrews within patients, it could not overcome the limited number of events. These regression findings should therefore be interpreted as exploratory and hypothesis-generating rather than confirmatory.

Patient acceptance was generally favourable: 78% of participants were satisfied or very satisfied, and 92% would recommend the procedure. These are patient-level findings and should remain analytically distinct from screw-level success and complication outcomes. Willingness to recommend the procedure does not necessarily indicate absence of discomfort or complications, because patients may accept short-term adverse experiences in view of the expected orthodontic benefit. Satisfaction may also have been influenced by the ongoing clinical relationship between participants and the treating investigator and by social-desirability bias. The findings therefore indicate favourable acceptance within this clinical setting rather than universal acceptability.

The principal strengths of this study include its prospective design, consecutive recruitment, predefined pain assessments, integration of patient-reported and clinical outcomes within the same cohort, separation of patient-level and screw-level analyses, and use of GEE to account for within-patient clustering. The pain-item-excluded sensitivity analysis also addressed potential direct mathematical overlap between pain and the oral-impact score.

Several limitations should be acknowledged. The study was conducted in a single private dental centre, and all procedures, clinical assessments, and outcome classifications were undertaken by one investigator. Operator-related measurement bias, institutional selection bias, and social-desirability bias therefore cannot be excluded, limiting generalisability to other operators, centres, populations, and clinical protocols. Complete-case analysis may also have introduced selection bias if participants with incomplete follow-up differed systematically from those analysed.

Pain reporting was subjective and may have been influenced by individual pain thresholds, anxiety, expectations, and previous dental experiences. Analgesic exposure was incompletely characterised, leaving potential residual confounding. The oral-impact questionnaire was administered only at one week and retrospectively assessed experiences during the first postoperative week; it has also not undergone full psychometric validation. Consequently, correlations between pain and oral-impact scores may have been influenced by recall bias, common-method bias, temporal overlap, and measurement uncertainty. Excluding the pain item reduced direct part–whole correlation but did not eliminate these other potential sources of association. Therefore, neither causality nor temporal direction can be established. The small number of failures limited the precision and stability of the regression model, while potentially relevant procedural variables—including insertion torque, insertion angle, cortical bone characteristics, and the magnitude and timing of orthodontic loading—were not systematically recorded. Finally, the six-month observation period evaluated short- to medium-term performance but did not establish longer-term stability.

The findings support informing patients that discomfort is generally concentrated in the early postoperative period and decreases substantially within one week. Patients reporting greater early pain may also report less favourable short-term oral impacts and may warrant closer clinical review and appropriate postoperative support. However, the study did not test whether any particular analgesic regimen, counselling approach, or postoperative intervention improves oral-impact scores, satisfaction, or miniscrew success. Multicentre studies incorporating independent outcome assessment, standardised documentation of analgesic and procedural variables, longer follow-up, and comprehensive psychometric validation of the questionnaire are warranted.

## 5. Conclusions

In this single-centre cohort, pain decreased substantially during the first postoperative week, and greater early pain was associated with less favourable scores on a preliminary miniscrew-related oral-impact questionnaire. A six-month miniscrew success rate of 92.6% and favourable patient acceptance were observed under the study protocol. The questionnaire findings and exploratory failure model require confirmation in larger, multicentre studies using fully validated patient-reported measures.

## Figures and Tables

**Table 1:** Miniscrew success by anatomical location.

Site	Number (%)	Number of Failures	Success Rate (%)
Upper premolar region	23 (8.5%)	1	95.7%
Upper molar region	173 (64.1%)	13	92.5%
Lower premolar region	26 (9.6%)	1	96.2%
Lower molar region	48 (17.8%)	5	89.6%
Total	270 (100%)	20 (7.4%)	92.6%

**Table 2:** Preliminary reliability estimates for the miniscrew-related oral-impact questionnaire.

Measure	Sample	Estimate	95% CI	*p*-Value
Cronbach’s alpha (α, *N* = 100)	*N* = 100	0.716	-	-
McDonald’s omega (ω, *N* = 100)	*N* = 100	0.803	-	-
Test–retest difference (*p*-value)	*n* = 10	-	-	0.642
Intraclass correlation coefficient [ICC(2,1)]	*n* = 10	0.92	0.72–0.98	*p* < 0.001

*Note*. Cronbach’s alpha (α) and McDonald’s omega (ω) were calculated in the main sample (*N* = 100); the intraclass correlation coefficient ICC and the Wilcoxon signed-rank test were calculated in the pilot sample (*n* = 10).

**Table 3:** Distribution of responses and item scores for the preliminary eight-item miniscrew-related oral-impact questionnaire.

Domain	Item Question	Never *n* (%)	Rarely *n* (%)	Sometimes *n* (%)	Often *n* (%)	Always *n* (%)	Mean ± SD	Median (IQR)
Physical	1. Pain or discomfort in the area of the screw.	63 (63.0%)	11 (11.0%)	9 (9.0%)	12 (12.0%)	5 (5.0%)	0.85 ± 1.28	0.00 (0.00−2.00)
	2. Irritation of lips or cheeks.	39 (39.0%)	13 (13.0%)	17 (17.0%)	16 (16.0%)	15 (15.0%)	1.55 ± 1.51	1.00 (0.00−3.00)
Functional	3. Difficulty chewing or eating.	58 (58.0%)	5 (5.0%)	17 (17.0%)	7 (7.0%)	13 (13.0%)	1.12 ± 1.49	0.00 (0.00−2.00)
	4. Difficulty speaking clearly.	72 (72.0%)	6 (6.0%)	9 (9.0%)	11 (11.0%)	2 (2.0%)	0.65 ± 1.15	0.00 (0.00−1.00)
Psychological	5. Feeling embarrassed about the screw or its visibility.	79 (79.0%)	5 (5.0%)	3 (3.0%)	3 (3.0%)	10 (10.0%)	0.60 ± 1.30	0.00 (0.00−0.00)
	6. Feeling less confident during treatment.	84 (84.0%)	5 (5.0%)	5 (5.0%)	3 (3.0%)	3 (3.0%)	0.36 ± 0.94	0.00 (0.00−0.00)
Social	7. Avoiding smiling or social interactions.	85 (85.0%)	3 (3.0%)	4 (4.0%)	4 (4.0%)	4 (4.0%)	0.39 ± 1.02	0.00 (0.00−0.00)
	8. Sleep disturbances due to screw discomfort.	73 (73.0%)	11 (11.0%)	7 (7.0%)	5 (5.0%)	4 (4.0%)	0.56 ± 1.09	0.00 (0.00−1.00)
Total oral-impact score		—	—	—	—	—	6.08 ± 5.92	5.00 (1.00−9.25)
Modified seven-item oral-impact score (Excluding Item 1)							5.23 ± 5.24	3.50 (1.00−8.00)

**Table 4:** Spearman rank correlations between pain intensity and miniscrew-related oral-impact scores.

Pain Assessment (Time Point)	Spearman’s Rho (*ρ*)	95% CI	Holm-Adjusted *p*-Value	Pain-Excluded (Item 1 Excluded) (*ρ*)	95% CI	Exploratory *p*
During Placement	0.431	0.256 to 0.578	<0.001 ***	0.395	0.215 to 0.549	*p* < 0.001 ***
At 24 Hours	0.297	0.107 to 0.466	0.003 **	0.331	0.144 to 0.495	*p* < 0.001 ***
At One Week	0.312	0.123 to 0.479	0.002 **	0.346	0.160 to 0.508	*p* < 0.001 ***

*Note.* To rule out artificial inflation (item-overlap bias) caused by including procedural pain within both the pain predictor variables and the total minscrew-related oral impact score, a sensitivity analysis was performed using the modified seven-item oral-impact score (excluding Item 1) ** *p* < 0.01; *** *p* < 0.001.

**Table 5:** Exploratory multivariable logistic GEE analysis of factors associated with miniscrew failure, accounting for clustering of 270 miniscrews within 100 patients.

Factor	Adjusted OR	Robust 95% CI	*p*-Value
Anatomical arch (maxilla vs. mandible)	0.63	[0.22, 1.82]	0.386
Miniscrew length (per mm increase)	0.92	[0.77, 1.09]	0.315
Miniscrew diameter (per mm increase)	0.42	[0.03, 5.76]	0.551
Patient age (per one-year increase)	1.01	[0.95, 1.08]	0.654

Note: OR, odds ratio; CI, confidence interval; GEE, generalised estimating equation. The model used a binomial distribution, logit link, exchangeable working correlation structure, patient identifier as the clustering variable, and robust sandwich standard errors. The four predictors were selected a priori on clinical grounds and entered simultaneously without automated variable selection. Because only 20 failures occurred—approximately five events per estimated predictor coefficient—the estimates are exploratory and potentially imprecise; interpretation should emphasise the robust 95% confidence intervals.

## Data Availability

The data supporting the findings of this study are available from the corresponding author upon reasonable request. The data are not publicly available because of patient confidentiality and ethical restrictions.

## Abbreviations

The following abbreviations are used in this manuscript:

TAD	Temporary anchorage device
VAS	Visual analogue scale
OHRQoL	Oral health-related quality of life
OHIP	Oral Health Impact Profile
ICC	Intraclass correlation coefficient
IQR	Interquartile range
OR	Odds ratio
CI	Confidence interval
GEE	Generalised estimating equation
